# Investigation of Antimicrobial Potential of Medicinal Plants Against *Pseudomonas aeruginosa*

**DOI:** 10.1002/fsn3.70999

**Published:** 2025-11-14

**Authors:** Nosheen Bibi, Shazia Perveen, Sumaira Kanwal, Fariha Latif, Rehmana Rashid, Sara Janiad, Iram Qadeer, Fatima Naseem, Abdullah R. Alanzi, Rashed N. Herqash, Imran Haider, Mehraj A. Abbasov, Sadaf Kayani, Mohd Asif Shah

**Affiliations:** ^1^ Department of Zoology The Women University Multan Multan Pakistan; ^2^ Department of Biosciences COMSATS University Islamabad Sahiwal Pakistan; ^3^ Institute of Zoology Bahauddin Zakariya University Multan Multan Pakistan; ^4^ Department of Pharmacy The Women University Multan Multan Pakistan; ^5^ Department of Microbiology and Molecular Genetics The Women University Multan Multan Pakistan; ^6^ Department of Zoology The Government Sadiq College Women University Bahawalpur Pakistan; ^7^ Goulburn Valley Health, Graham St. Shepparon Shepparton Australia; ^8^ Center for Research on Medicinal, Aromatic and Poisonous Plants, DSR King Saud University Riyadh Saudi Arabia; ^9^ National Research Center of Intercropping The Islamia University of Bahawalpur Bahawlp Pakistan; ^10^ Scientific Research Centre Azerbaijan Medical University Baku Azerbaijan; ^11^ International Center for Biodiversity and Genomics Baku Eurasian University Baku Azerbaijan; ^12^ Department of Biology, Faculty of Engineering and Natural Science Suleyman Demiral University Isparta Turkiye; ^13^ Kardan University Kabul Afghanistan; ^14^ Division of Research and Development Lovely Professional University Phagwara India; ^15^ University Centre for Research and Development Chandigarh University Mohali India

**Keywords:** antimicrobial activity, medicinal plants, oral health, phytochemicals, *Pseudomonas aeruginosa*

## Abstract

*Pseudomonas aeruginosa*, a gram‐negative bacterium present in water and soil, causes significant illnesses and chronic conditions that degrade drinking water quality. This study aimed to evaluate the antibacterial and antioxidant activities of plant extracts (*Convolvulus arvensis*, *Chenopodium murale*, *Avena fatua*, *Cirsium arvense*, and *Hordeum vulgare*) against *P. aeruginosa*. FTIR spectroscopy was used to investigate in the plant extracts, and the phytochemical compounds found in the plant extracts were analyzed using GC–MS analysis. This study evaluated the antioxidant activity of plant extracts using DPPH scavenging assays and found that ascorbic acid significantly improved the DPPH scavenging activity of *C. arvensis* extract compared to that of other plants. Saliva samples were collected from the patients to differentiate *P. aeruginosa* from other oral cavity microorganisms. Gram staining and catalase testing were performed to identify bacterial strains. *Pseudomonas aeruginosa* produces a positive catalase test and appears as a pink or red rod after Gram staining. This study revealed that five plant extracts have antibacterial activity against *P. aeruginosa. Cirsium arvensis* showed a higher zone of inhibition (72 mm). The MIC was the lowest concentration that inhibited the visible growth of a bacterium, whereas the MBC was the lowest concentration that killed 99.9% of the bacteria. *A. fatua* and *C. murale* were effective at very low concentrations, inhibiting bacterial growth at 1 μg/mL and killing bacteria at 2 μg/mL. *Hordeum vulgare* and *C. arvense* require slightly higher concentrations to effectively inhibit bacterial growth and to exert bactericidal effects. All the studied plant extracts were effective in inhibiting biofilm formation by *P. aeruginosa*; however, *H. vulgare* demonstrated the highest percentage of inhibition (99.05) at a concentration of 0.8 μg/mL. This study revealed that these plants exhibit antibacterial activity against *P. aeruginosa*.

## Graphical abstract

This study evaluated the antibacterial and antioxidant activities of five plant extracts against *Pseudomonas aeruginosa*. The results revealed significant antibacterial effects, with *Cirsium arvense* showing the highest inhibition zone and *Horedeum vulgare* exhibiting strong biofilm inhibition, highlighting the potential of these plants in antimicrobial applications.

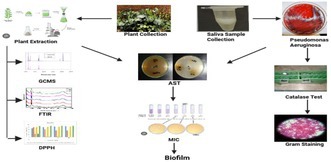

## 1 Introduction

Infectious diseases have increased significantly in economically developed countries in recent years. They have become the second‐largest cause of mortality globally and the third most important cause of death (De Zoysa et al. 2019). *Pseudomonas aeruginosa* is one of the most important opportunistic human pathogens, specifically *P. aeruginosa*. Patients suffering from different infections, like cystic fibrosis, immunocompromised patients, chronic obstructive pulmonary diseases, and AIDS (Acquired Immunodeficiency Syndrome) patients, are infected by it (Ganesh and Rai 2018). *Pseudomonas aeruginosa* causes a wide variety of infections owing to its extensive arsenal of virulence factors, which include enzymes such as proteases and elastases, phenazine pigments, and numerous export mechanisms involved in virulence secretion (Kerr and Snelling 2009). *Pseudomonas aeruginosa* is a gram‐negative bacterium that is commonly found in water and soil (Al‐Daghistani, Abu‐Niaaj, and Zein 2025; Bahmani et al. 2016). It is known to cause serious infections and chronic disorders and can also impair the quality of drinking water. The World Health Organization (WHO) and European Communities have identified it as an indicator of drinking water quality. Early detection is critical for treatment using modern techniques, such as flow cytometry (Tang et al. 2017).

In a prior investigation, from hospitalized patients, *P. aeruginosa* was recovered from nosocomial pneumonia (16%), post‐operative wound infections (85%), hospital blood infections (10%), nosocomial urinary tract infections (12%), and accounting for 23% of the total isolated germs (Al‐Daghistani, Matalqah, et al. 2025; Bahmani et al. 2016). Previous research has revealed the risk factors for Pseudomonas resistance, such as increased death rates and fluoroquinolone use (Shang et al. 2024). Controlling resistance is critical because of the scarcity of alternative medications for gram‐negative bacteria (Goldstein et al. 2009). Over the last two decades, the antibacterial characteristics of medicinal plants have been extensively studied in plant components, such as leaves, seeds, and fruits. Antibiotic principles are extensively distributed in angiospermic plants and medicinal plant extracts that are high in antibacterial chemicals and can be employed to remove microorganisms from edible products (Alagesaboopathi 2011).

The World Health Organization reports that approximately 80% of the population in developing regions continues to rely on traditional medicines derived from medicinal plants for primary healthcare needs. Globally, around 374,000 plant species have been identified, yet only about 28,187 species are known to be used by humans for medicinal or other purposes (Vaou et al. 2021). Over 20,000 species have antimicrobial activity, and more than 30,000 compounds have been isolated from plants (Srinivasan et al. 2001). 14%–28% of higher plant species are medicinal (Pandey and Shashank Kumar 2013). Hippocrates' famous quote, “The true healers of disease are Natural forces,” captures the idea that nature was the only unbounded source of therapeutic ingredients for thousands of years before the discovery of synthetic medications (Zhang et al. 2022). The vital goal is to use natural products for therapeutic purposes that have beneficial antibacterial, anti‐inflammatory, and anticancer characteristics and no negative side effects at the selected concentration. As a result, we have recently observed a variety of plant extracts and unadulterated natural components used in everyday medicine as part of several treatment regimens (Hickl et al. 2018).

The main purpose of this study was to test how extracts from five medicinal plants (*Convolvulus arvensis*, *Avena fatua*, *Cirsium arvense*, *Chenopodium murale*, and *Hordeum vulgare*) kill or inhibit the growth of *P. aeruginosa*. We aimed to explore the potential of these plants as natural germ killers and to enhance human health.

## 2 Material and Method

### 2.1 Plants Collection

Five plants (*C. arvensis*, *A. fatua*, *C. arvense*, *C. murale*, and *H. vulgare*) were used in this study. The plants examined were purchased from a local store in Multan, Pakistan. The collected plants were identified, and the nomenclature was confirmed by a plant taxonomist. Each raw item was washed with cold, fresh tap water, filtered through sterilized distilled water, and then left to air dry. All dried plants were ground into a powder using a sterile grinding device. The extracted powder was then immediately subjected to the same procedure.

### 2.2 Plants Extraction

Plant extracts (*C. arvensis*, *A. fatua*, *C. arvense*, *C. murale*, and *H. vulgare*) were prepared by combining 10 g dried crushed material with 50 mL ethanol. The solutions were then gradually mixed on an orbital shaker for 72 h. To obtain clear and pure extracts, the mixtures were filtered through Whatman filter paper. A rotary evaporator was then used to dry each mixture, yielding a dry crude powder extract. The crude extracts were dissolved in the minimum volume of ethanol (1 mL) after filtration to prepare the final stock solutions, which were then stored at 4°C in sealed, sterile falcon tubes.

### 2.3 Fourier‐Transformed Infrared (FTIR) Spectroscopic Analysis

Potassium bromide (KBr) discs were used to perform FTIR spectroscopy on ethanolic plant extracts. To make the disc, we mixed 1 mg of the sample and 100 mg of KBr and then subjected it to a dye that was compressed using a hydraulic press. This was done to obtain FTIR spectra in the 4000–400 cm^−1^ range. We then analyzed the spectra to identify the functional groups present in the plant samples (Saleem, Zaib, et al. 2020).

### 2.4 Gas Chromatography–Mass Spectroscopy Analysis

Plant extracts (*C. arvensis*, *A. fatua*, *C. arvense*, *C. murale*, and *H. vulgare*) were chemically analyzed using GC–MS. The column DB5 Durabond utilizes fused silica. The dimensions of the column (DB5) are 30 m × 0.25 mm ID, and the film thickness is 0.25 μm. The temperature settings for the oven were 80°C (10°C/min), 200°C (12°C/min), and 260°C for 30 min. We adjusted the ion‐source temperature to 280°C and used helium gas (99.999%) as the carrier gas, which was constantly pumped at a rate of 1 mL per minute. We set the injection volume at 1 μL (10:1) and the injector temperature at 250°C and ranged the compound amu values from 50 to 550. The molecular weights and structures of the compounds in the test materials were determined using the National Institute of Standards and Technology (NIST) database after analyzing the GC–MS mass spectrum (Promprom and Chatan 2017).

### 2.5 DPPH Scavenging Activity

We assessed DPPH using an earlier process. Three milliliters of various quantities of plant extract were combined with 1 mL of freshly prepared methanol solution containing 0.004% DPPH. The mixed solutions were then incubated in the dark for 30 min. Absorbance was then measured at 517 nm. The low absorbance of the mixture indicated strong radical scavenging activity. The antioxidant activity of ascorbic acid has also been established. The control group was a solvent that did not contain any plant extract. Each experiment was performed three times (Saleem, Zaib, et al. 2020).

$$\text{DPPH inhibition percent}=\frac{\left(\text{Absorbance of control}-\text{Absorbance of the sample}\right)}{\text{Absorbance of control}\times 100}$$

### 2.6 Sample Collection

This is certified that patients' proforma‐based ethical permission and informed consent was obtained for the collection of saliva samples from patients and the study was carried out in compliance with guidelines of EU Directive 2001/20/EU for human experiments and under guidelines of the National Research Council's Guide for the Care and Use of humans and received approval from the Ethical Committee. The Women's University Multan under the approval number IRB number: WUM/REC/24‐20. This is to certify that there are no human images, videos, names, or any other form of human identification included in this study, which was conducted in accordance with the ethical guidelines of the Declaration of Helsinki.

Sampling was conducted at Bakhtawar Amin Medical and Dental College, Multan. Fifty saliva samples were collected specifically from patients diagnosed with dental caries, as opportunistic pathogens such as *P. aeruginosa* have been increasingly reported in individuals with compromised oral health. This patient group was selected to increase the likelihood of *P. aeruginosa*. Saliva samples from healthy individuals were not included. All bacterial isolates were confirmed to be *P. aeruginosa* based on colony morphology, Gram staining, and biochemical tests. Patients with diseases other than dental caries were excluded from the study.

To isolate the bacteria, saliva samples were collected under sterile conditions and placed in 2 mL autoclaved Eppendorf tubes. Next, Eppendorf tubes were placed within ice packs to prevent temperature fluctuations during sample transportation and to maintain the temperature of the samples. Before performing further testing, samples were carried from The Women University Multan's Department of Microbiology and Molecular Genetics Research Lab and kept at 4°C.

### 2.7 Culturing Bacterial Strains

#### 2.7.1 Blood Agar Preparation

Blood agar was used to cultivate bacterial strains obtained from dental caries saliva samples. It was created by combining 100 mL nutrient agar with 10 mL blood. All components were added to distilled water and gently swirled until the liquid was well combined and all ingredients were dissolved to create nutrient agar, also known as N‐agar. After autoclaving for 15 min at 121°C, N‐agar was allowed to cool to 45°C–50°C while it was still liquid. Nutrient agar was then mixed with 10 mL of blood to prevent the formation of air bubbles. Subsequently, sterile Petri dishes were filled with the combined blood agar medium and allowed to set. Petri dishes were then stored at 4°C until saliva samples were obtained.

1. *Isolation of bacterial strain*: Saliva samples were then swabbed onto blood agar using sterile cotton swabs in a biosafety cabinet (BSC) to avoid contaminating Petri plates and samples and then incubated at 37°C for 18 to 24 h.
2. *Purification of bacterial strains on L‐agar*: Bacterial strains were subcultured on **L** Agar (HiMedia M001‐500G) medium to produce pure colonies and then placed in an incubator at 37°C for 18 to 24 h.

### 2.8 Gram Staining Procedure

Gram staining was used to determine the identity of the bacteria. Utilizing light microscopy Model: AY13116, the cell morphology and size were examined. This approach distinguishes between gram‐positive and gram‐negative bacteria. The primary uses of this method are to determine the size and form of the cells. The selected culture strains were analyzed under an oil immersion lens using a light microscope after Gram staining. Bacterial strains from a 24‐h‐old culture were treated using this staining technique, and the germs were then allowed to develop on blood agar at 37°C.

1. The smear was produced on a clean glass slide and solidified via heating.
2. The second step involved adding crystal violet dye and letting it sit on the slide for a minute.
3. The slide was cleaned with tap water after Crystal Violet (CV) staining.
4. Subsequently, iodine solution was added, and the CV stain was allowed to react for a minute.
5. The running tap was used to clean the slide one more time.
6. The slide was then submerged three to five times in decolorizing solution to ensure that there was no primary stain.
7. The slide was cleaned again under running water and then allowed to air dry after adding safranin and letting it sit for a minute.
8. The morphology of each slide that had been prepared in this way for bacterial strains was examined using a light microscope (Paray et al. 2023).

### 2.9 Catalase Test

The Catalase test was used to differentiate certain bacterial strains from other oral microorganisms in the saliva samples. Using a sterile loop of metal wire, a small colony from a fresh culture was selected for this experiment and placed on a dry, sterile glass slide. Next, a small drop of H_2_O_2_ (hydrogen peroxide) was introduced into the slide and observed carefully. The lack of catalase enzyme production caused the absence of bubble formation. As none of the tested bacterial strains possessed catalase activity, hydrogen peroxide (H_2_O_2_) remained undecomposed and was not converted into oxygen or water. Consequently, there is no visible oxygen bubbling (Hyder et al. 2020; Paray et al. 2023).

### 2.10 Antibacterial Susceptibility Test

The agar well diffusion method was used for antibacterial susceptibility testing. Antibacterial efficacy against *P. aeruginosa* was evaluated using ethanolic extracts of plant extracts. In a 90 mm sterile Petri plate, 1.0 mL of standardized inoculum was inoculated, and 19.0 mL of autoclaved Mueller Hinton agar was cooled to 45°C to ensure even mixing of the contents and shaken it for 1 min. The planted plates were allowed to harden. A sterile 6 mm cork borer was used in the wells that were punched on the agar plate, and 100 μL of different concentrations of each extract were placed into the holes. The negative control contained 40% ethanol, whereas the positive control contained 100 μL of pure distilled water in a separate hole. The extracts were allowed to diffuse into the medium before incubation at 37°C for 24 h. The antibacterial activity was assessed by measuring the diameter of the inhibition zone surrounding each well (Islam et al. 2012).

### 2.11 Minimum Inhibitory Concentration and Minimum Bactericidal Concentration

The minimum inhibitory concentration (MIC) was determined using a microplate dilution. To a concentration of 8 mg/mL, crude plant extracts were again dissolved in a mixture of 50% distilled water and 50% solvent, namely, acetone (AC). In a 96‐well microtiter plate, 100 μL of the plant extract and gentamicin (used as the positive control) were mixed with purified water. Each well plate received 100 μL of the bacterial suspension, which was standardized to McFarland standard no. 0.5. In these studies, plant extracts at the following quantities were used at concentrations of 2, 1, 0.5, 0.25, 0.125, 0.062, 0.031, and 0.015 mg/mL. For 24 h, wrapped plates were left to incubate at 37°C. Next, 40 μL p‐iodonitrotetrazolium violet was diluted in distilled water and placed in a well plate. The wells were then incubated at 37°C for 30 to 45 min to allow bacterial growth. A pink or red hue was used to indicate the presence of bacterial growth, whereas clear wells indicated that the plant extract inhibited bacterial development. Gentamicin (50 μg/mL) was used as a positive control, and extract‐dissolving solvents were used as negative controls. The experiment was carried out three times in quadruplicate (Nkala et al. 2019).

### 2.12 Biofilm Plate Assay

A clinical isolate of *pseudomonas* from saliva samples was cultured in MHB (Muller Hinton Broth) containing 1% sucrose (MHB‐S) at 37°C for the entire night in an aerobic environment with 5%–10% CO_2_. Next, 100 μL of MHB‐S, comprising 10 distinct concentrations (0.02–10 mg/mL) of the examined plant extracts, was applied to each of the 96‐well polystyrene tissue‐culture plates. Additionally, 5 μL of the *pseudomonas* overnight culture was added to each well. A series of DMSO dilutions was investigated concurrently. The negative and positive bacterial growth controls consisted of wells containing only MHB‐S and a series of 0.2% chlorhexidine dilutions, respectively. The 96‐well plates were then placed in an aerobic environment containing 5%–10% CO_2_ and incubated at 37°C for 48 h. After discarding the culture media, non‐adherent bacteria were removed from the wells by washing three times with 300 mL of phosphate‐buffered saline per plate. After allowing the plates to air dry, they were stained for 10 min with Carbol Gentian Violet solution for microscopy, which contained 0.1%–0.25% methyl violet. To remove extra stain, the plates were rinsed with distilled water. After a 10‐min drying period at 60°C, the plates were resolubilized by adding 50 μL of absolute ethanol (99.9% v/v) containing the dye to each well for subsequent analysis. A Tecan Infinite 200 plate reader was used to detect optical density at 595 nm. Mean values were calculated and all experiments were performed in quadruplicate. Using two distinct cut‐off values, no biofilm production, or C1; moderate biofilm creation, C2; and high biofilm production, or C3, the anti‐biofilm effect of each plant extract on *pseudomonas* was grouped into three groups during the analysis. The negative control to estimate the low cut‐off value was increased by three standard deviations from the blank value. After three measurements of the low cutoff value, a high cutoff value was determined (Hickl et al. 2018).

## 3 Results

### 3.1 Extraction From Plants

Extracts were obtained from five plants (*C. arvensis*, *C. murale*, *H. vulgare*, *C. arvense*, and *A. fatua*) to determine their antimicrobial activity against the pathogenic bacterium, *P. aeruginosa*.

### 3.2 Analysis of FTIR Spectra

**FIGURE 1:**
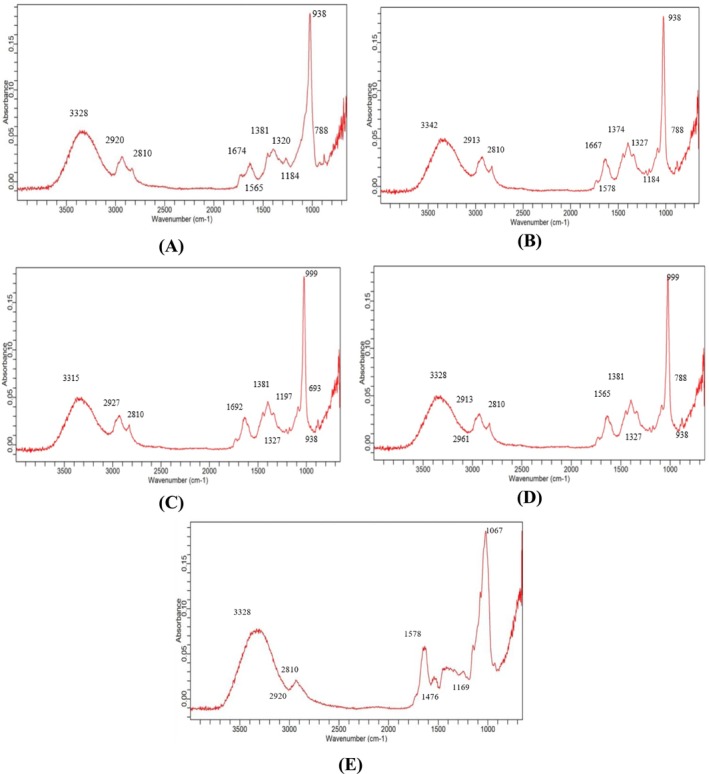
FTIR spectra of five plant extracts: (A) FTIR of *Convolvulus arvensis*, (B) FTIR of *Chenopodium murale*, (C) FTIR of *Avena fatua*, (D) FTIR of *Cirsium arvense*, and (E) FTIR of *Hordeum vulgare*.

FTIR spectroscopy is an advanced technique frequently used to examine bioactive substances in natural products. In the IR spectrum (Figure 1), individual peaks corresponding to a particular identified in each sample show functional groups. These wavenumbers are associated with different types of stretching vibrations, such as peaks at 788 and 2810 cm^−1^ showing C—H stretch (Adib and Abdullah 2018), peaks at 2961 and 2920 cm^−1^ showing CH and CH2 stretching vibrations, peaks at 3315 and 3328 cm^−1^ showing O—H stretching vibrations (Adib and Abdullah 2018; Joselin et al. 2014), peaks at 1184, 1258, 1327, 1197, and 1320 cm^−1^ showing S=O bond, peaks at 1565, 1578, 1592, 1667, and 1674 cm^−1^ showing C=O stretching vibrations (Maher et al. 2021), a peak at 693 cm^−1^ showing C—X stretching vibration, peaks at 938, 996, 999, 1067, and 1169 cm^−1^ showing C—O vibrations (Yin et al. 2018), and the peaks at 1381 and 1374 cm^−1^ showing C—C stretching vibrations (Kusumadewi et al. 2022).

### 3.3 Gas Chromatography–Mass Spectrometry

Different numbers of phytochemical compounds were identified in the five plant extracts. A total of 27 compounds were observed in the *C. arvensis*. In the extracts of *C. murale*, *A. fatua*, *C. arvense*, and *H. vulgare*, 55, 24, 33, and 19 compounds, respectively, were identified. The names of the compounds, retention time, molecular formula, molecular weight, and peak area percentages are presented in Tables 1, 2, 3, 4, 5, respectively. These findings align with those of previous studies employing GC–MS analysis, in which various bioactive compounds have been reported from plant extracts. For example, identified key constituents such as 1,2‐ethanediamine, N′‐ethyl‐N, N‐dimethyl‐ (36.01%) and 2,3‐butanediol, 1,4‐dimethoxy‐ in *C. arvensis* (Jalill et al. 2014).

**TABLE 1:** Phytochemicals detected in the extract of *Convolvulus arvensis* by GCMS analysis.

Peak no.	Phytochemical	Retention time	Molecular weight	Area (%)
1	1,6‐Anhydro‐2,4‐dideoxy‐.beta.‐D‐ribo‐hexopyranose (C_6_H_10_O_3_)	3.89	130.1	3.32
2	2‐Hydroxy‐gamma‐butyrolactone (C_4_H_6_O_3_)	3.99	102	1.1
3	Benzothiazole (C_7_H_5_NS)	8.11	135.1	3.59
4	1,4‐Dioxane‐2,6‐dimethanol (C_6_H_12_O_4_)	13.34	148.1	11.15
5	1,3‐Dioxane‐5‐methanol, 5‐ethyl‐(C_7_H_14_O_3_)	13.65	146.1	17.66
6	Bis(1‐methylheptyl) methylphosphonate (C_17_H_37_O_3_P)	14.25	320.2	1.09
7	2,4‐Di‐tert‐butylphenol (C_14_H_22_O)	14.46	206.3	2.28
8	4‐Bromo‐N‐(thiophen‐2‐ylmethyl) aniline (C_11_H_9_BrFNS)	18.77	286.1	0.91
9	3, 5‐di‐tert‐Butyl‐4‐hydroxybenzaldehyde (C_15_H_22_O_2_)	20.15	234.3	0.4
10	1,2‐Benzenedicarboxylic acid, monobutyl ester (C_12_H_14_O_4_)	22.28	222.2	1.31
11	4‐Acetylbenzoic acid (C_9_H_8_O_3_)	22.41	164.1	0.14
12	Hexadecanoic acid, methyl ester (C_17_H_34_O_2_)	23.59	270.4	6.87
13	Tridecanoic acid (C_13_H_26_O_2_)	24.25	214.3	1.62
14	4,4‐Dimethyl‐oct‐5‐enal (C_10_H_18_O)	24.32	154.2	0.6
15	Hexadecanoic acid, ethyl ester (C_18_H_36_O)	24.91	284.4	1.38
16	2‐Methyl‐6‐nitroindolizine (C_7_H_8_N_2_O_2_)	26.8	152.1	1.63
17	Pentyl dotriacontyl ether (C_37_H_76_O)	27.09	537.0	1.82
18	Methyl 2‐hydroxy‐pentadecanoate (C_6_H_12_O_3_)	28.01	272.4	0.61
19	9, 12, 15‐Octadecatrienoic acid, ethyl ester (C_20_H_34_O_2_)	28.11	306.5	1.58
20	Phthalic acid, monoamide, N, N‐dicyclohexyl‐, ethyl ester (C_25_H_37_NO_3_)	28.67	399.6	1.25
21	Palmitoleic acid (C_16_H_30_O_2_)	28.87	254.4	0.66
22	N, N‐Dimethylpalmitamide (C_18_H_39_N)	29.64	269.5	1.57
23	1,2‐Benzisothiazole‐3‐acetic acid, methyl ester (C_10_H_9_NO_2_S)	31.77	207.2	0.9
24	Phthalic acid, di (2‐propylpentyl) ester (C_26_H_26_O_4_)	34.17	402.5	15.04
25	1‐(3‐Chlorophenyl)‐3‐methyl‐1H‐pyrazol‐5‐amine (C_10_H_10_ClN_3_)	36.37	**283.7**	0.71
26	1,4‐Benzenedicarboxylic acid, bis (2‐ethylhexyl) ester (C_24_H_38_O_4_)	37.3	390.5	5.3
27	2‐Ethylacridine (C_15_H_13_N)	40.56	207.2	1.5

**TABLE 2:** Phytochemicals detected in the extract of *Chenopodium murale* by GCMS analysis.

Peak no.	Phytochemical	Retention time	Molecular weight	Area (%)
1	Succinic acid, 2‐chloropropyl dodecyl ester (C_19_H_35_ClO_4_)	3.91	362.9	0.07
2	Cyclohexene, 3,5,5‐trimethyl‐(C_9_H_16_)	6.84	124.2	0.08
3	Cyclohexanol, 3,3,5‐trimethyl‐, acetate, cis‐(C_11_H_20_O_2_)	6.96	184.2	0.27
4	Cyclohexanecarboxylic acid, 4‐pentyl‐, 4‐fluorophenyl ester (C_19_H_28_O_3_)	11.48	304.4	1.69
5	2‐Thiopheneacetic acid, 2,7‐dimethyloct‐7‐en‐5‐yn‐4‐yl ester (C_16_H_20_O_2_S)	12.52	276.3	0.32
6	Thiophene‐2‐acetic acid, dodec‐9‐ynyl ester (C_18_H_26_O_2_S)	12.85	306.5	0.56
7	3‐Cyclopent‐1‐enyl‐3‐hydroxy‐2‐methylpropionic acid (C_9_H_14_O_3_)	12.99	170.2	0.68
8	Octadecane, 3‐ethyl‐5‐(2‐ethylbutyl) ‐(C_26_H_54_)	13.37	366.7	0.17
9	2,4‐Di‐tert‐butylphenol (C_14_H_22_O)	13.51	206.3	0.74
10	1,1‐Dimethyl‐1‐silacyclo‐3‐pentene (C_6_H_12_Si)	13.91	112.2	0.31
11	2H‐Pyran‐2‐one, tetrahydro‐4‐(2‐methyl‐1‐propen‐3‐yl) ‐(C_9_H_14_O_2_)	14.16	154.2	0.38
12	Methylphosphonic acid, di (1‐methylpropyl) ester (C_5_H_12_O_3_S)	14.25	152.2	0.8
13	2,4‐Di‐tert‐butylphenol (C_14_H_22_O)	14.47	206.3	0.74
14	Hexadecane (C_16_H_34_)	16.15	226.4	0.14
15	Oxalic acid, cyclohexylmethyl tetradecyl ester (C_23_H_42_O_4_)	18.42	382.6	0.59
16	Heptadecane (C_17_H_36_)	18.64	240.4	0.26
17	Tridecanoic acid, 12‐methyl‐, methyl ester (C_15_H_30_O_2_)	19.19	242.3	0.19
18	Methyl tetradecanoate (C_15_H_30_O)	19.42	242.4	0.16
19	3, 5‐di‐tert‐Butyl‐4‐hydroxybenzaldehyde (C_15_H_22_O_2_)	19.91	234.3	0.14
20	Methyl 2,6‐dimethoxyisonicotinate (C_9_H_11_NO_2_)	20.26	165.1	0.12
21	Octadecane (C_18_H_38_)	20.9	254.4	0.24
22	Cyclopentadecane (C_15_H_30_)	21.79	210.3	0.11
23	Nonyl tetradecyl ether (C_23_H_48_O)	21.91	340.6	0.09
24	Ethanone, 1,2‐di‐2‐furanyl‐2‐hydroxy‐(C_10_H_8_O_4_)	22.14	192.1	0.14
25	3‐(But‐3‐enyl)‐cyclohexanone (C_10_H_16_O)	22.27	152.2	0.15
26	Fumaric acid, 4‐bromophenyl cyclohexylmethyl ester (C_17_H_19_BrO_4_)	22.65	367.2	0.17
27	Nonadecane (C_19_H_40_)	23.02	268.5	0.46
28	9‐Hexadecenoic acid, methyl ester, (Z)‐(C_17_H_32_O)	23.42	268.4	0.13
29	Hexadecanoic acid, methyl ester (C_17_H_34_O_2_)	23.53	270.4	5.81
30	Succinic acid, tridec‐2‐yn‐1‐yl cis‐4‐methylcyclohexyl ester (C_24_H_40_O_4_)	23.94	392.5	0.1
31	Sulfurous acid, cyclohexylmethyl hexyl ester (C_13_H_26_O_3_S)	24.24	262.4	1.28
32	Heptane, 1,7‐dibromo‐(C_7_H_14_Br_2_) _	25.15	257.9	0.63
33	Succinic acid, cyclohexylmethyl 4‐acetylphenyl ester (C_19_H_24_O_5_)	25.21	332.3	0.71
34	Hexadecanoic acid, 14‐methyl‐, methyl ester (C_18_H_36_O_2_)	25.53	284.2	0.19
35	Cyclodecasiloxane, eicosamethyl‐ (C_20_H_60_O_10_Si_10_)	26.28	741.5	0.15
36	Octadecadienoic acid, methyl ester (C_19_H_34_O_2_)	26.78	294.5	1.18
37	Methyl stearate (C_19_H_38_O_2_)	27.41	298.5	3.77
38	Methyl 8‐(5‐hexyl‐2‐furyl)‐octanoate (C_19_H_32_O_3_)	27.84	308	0.63
39	2′‐Hydroxy‐5′‐methoxyacetophenone, isopropyl ether (C_12_H_16_O_3_)	27.93	208.2	0.59
40	Linoleic acid ethyl ester (C_20_H_36_O_2_)	28.01	308.4	0.3
41	9,12,15‐Octadecatrienoic acid, ethyl ester (C_20_H_34_O_2_)	28.11	306.5	0.47
42	Methyl 2‐octylcyclopropene‐1‐heptanoate (C_19_H_34_O_2_)	28.52	294.4	0.17
43	1,3‐Dimethyl‐5‐ethyladamantane (C_14_H)	28.82	192.3	0.44
44	2‐Allylpent‐4‐enoic acid, methyl ester (C_9_H_14_O_2_)	29.37	154.2	0.2
45	Sebacic acid, di(4‐heptyl) ester (C_24_H_46_O)	29.47	398.6	1.28
46	2,4‐Heptadienoic acid, 6‐methyl‐, ethyl ester (C_10_H_16_O_2_)	29.87	168.2	0.41
47	Oxiraneoctanoic acid, 3‐octyl‐, methyl ester (C_18_H_34_O)	30.1	298.4	0.47
48	9‐Hexadecenoic acid, methyl ester, (9Z) (C_17_H_32_O_2_)	30.84	268.4	0.53
49	Eicosanoic acid, methyl ester (C_21_H_42_O)	30.92	326.5	0.55
50	3‐(But‐3‐enyl)‐cyclohexanone (C_10_H_16_O)	31.74	152.2	0.18
51	Octadecanoic acid, 9,10‐dihydroxy‐, methyl ester (C_19_H_38_O)	33.51	330.5	0.19
52	Phthalic acid, di (2‐propylpentyl) ester (C_26_H_26_O_4_)	34.17	402.5	1.34
53	Cyclobarbital (C_12_H_16_N_2_O_3_)	34.78	236.2	0.29
54	3‐Methoxy‐2,4,5‐trifluorobenzoic acid, nonadecyl ester (C_27_H_43_F_3_O_3_)	36	472.6	0.09
55	1,4‐Benzenedicarboxylic acid, bis (2‐ethylhexyl) ester (C_24_H_38_O_4_)	37.3	390.6	0.28

**TABLE 3:** Phytochemicals detected in the extract of *Avena fatua* by GCMS analysis.

Peak no	Phytochemical	Retention time	Molecular weight	Area (%)
1	Tetradecane (C_14_H_30_)	11.95	198.3	1.84
2	Bis(1,3‐dimethylbutyl) methylphosphonate (C_13_H_29_O_3_P)	14.25	264.3	2.03
3	2,4‐Di‐tert‐butylphenol (C_14_H_22_O)	14.47	206.3	5.49
4	Bis(4‐tert‐butyldimethylsiloxy) benzyl ether (C_26_H_42_O_3_Si_2_)	15.32	458.2	1.54
5	Hexadecane (C_16_H_34_)	16.67	226.4	1.92
6	Oxalic acid, cyclohexylmethyl tetradecyl ester (C_23_H_42_O_4_)	18.78	382.6	12.59
7	Heptadecane (C_17_H_36_)	18.9	240.4	1.32
8	3, 5‐di‐tert‐Butyl‐4‐hydroxybenzaldehyde (C_15_H_22_O_2_)	28.15	234.3	1.24
9	Octadecane (C_18_H_38_)	21.05	254.4	1.03
10	Pentadecanoic acid, 14‐methyl‐, methyl ester (C_17_H_34_O_2_)	23.59	270.4	2.63
11	Benzenepropanoic acid, 3,5‐bis(1,1‐dimethylethyl)‐4‐hydroxy‐, methyl ester (C_18_H_28_O_3_)	23.66	292.2	1.93
12	Hexadecanoic acid, ethyl ester (C_18_H_36_O_2_)	24.91	284.4	1.61
13	Octadecane (C_18_H_38_)	25.07	254.4	1.32
14	8‐Hexadecyne (C_16_H_30_)	26.67	222.4	1.61
15	Methyl stearate (C_19_H_38_O_2_)	27.42	298.5	1.5
16	Benzene, 1‐(1‐carboxyethyl)‐4‐(1‐formylethyl) ‐(C_12_H_14_O_3_)	28.67	245.2	1.23
17	2‐Dodecen‐1‐yl (−) succinic anhydride (C_16_H_26_O_3_)	30.54	266.3	0.79
18	Phthalic acid, di (2‐propylpentyl) ester (C_26_H_26_O_4_)	34.17	279	21.7
19	Cyclopentanecarboxylic acid, hexadecyl ester (C_22_H_42_O_2_)	34.63	338.6	2.48
20	Propanamide, N‐(4‐methoxyphenyl)‐2,2‐dimethyl‐ (C_12_H_17_NO_2_)	36.02	207.2	0.64
21	1H‐imidazole‐2‐methanol, 1‐decyl‐(C_14_H_26_N_2_O)	36.75	238.3	1.1
22	1,4‐Benzenedicarboxylic acid, bis (2‐ethylhexyl) ester (C_24_H_38_O_4_)	37.3	390.5	6.08
23	4‐Methyl‐2‐trimethylsilyloxy‐acetophenone (C_12_H_18_O_2_Si)	38.18	222.3	2.54
24	5‐Methyl‐2‐phenylindolizine (C_15_H_13_N)	39.02	207.2	0.09

### 3.4 DPPH Scavenging Activity

**FIGURE 2:**
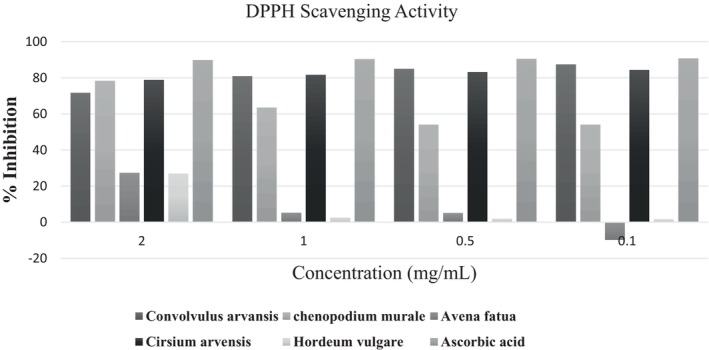
DPPH scavenging activity.

Antioxidant activity was measured by performing DPPH scavenging assays, and the plant extracts showed varying DPPH capabilities with the standard ascorbic acid (Figure 2).

**TABLE 4:** Phytochemicals detected in the extract of *Cirsium arvense* by GCMS analysis.

Peak no.	Phytochemical	Retention time	Molecular weight	Area (%)
1	Tetradecane (C_14_H_30_)	11.45	198.3	1.12
2	3‐(But‐3‐enyl)‐cyclohexanone (C_10_H_16_O)	14.25	152.2	1.22
3	2,4‐Di‐tert‐butylphenol (C_14_H_22_O)	14.47	206.2	3.62
4	Heptasiloxane, hexadecamethyl‐(C_16_H_48_O_6_Si_7_)	15.32	533.1	1.79
5	Hexadecane (C_16_H_34_)	16.67	226.4	1.55
6	Dicyclohexyl methylphosphonate (C_13_H_25_O_3_P)	18.78	260.3	9.29
7	Dodecamethylcyclohexasiloxane (C_12_H_36_O_6_Si_6_)	18.83	444.9	2.6
8	Heptadecane (C_17_H_36_)	1891	240.4	1.11
9	3, 5‐di‐tert‐Butyl‐4‐hydroxybenzaldehyde (C_15_H_22_O_2_)	20.16	234.3	1.06
10	Octadecane (C_18_H_38_)	21.05	254.4	1.06
11	Cyclohexene, 1,6‐dimethyl‐(C_8_H_14_)	21.17	110.2	0.91
12	cis‐Dihydrocarvone (C_10_H_16_O)	21.79	152.2	0.89
13	Pentadecanoic acid, 14‐methyl‐, methyl ester (C_17_H_34_O_2_)	23.6	270.4	2.85
14	Benzenepropanoic acid, 3,5‐bis(1,1‐dimethylethyl)‐4‐hydroxy‐, methyl ester (C_18_H_28_O_3_)	23.67	292.2	1.47
15	Di‐n‐butylphthalate (C_16_H_22_O_4_)	24.18	278.3	0.97
16	2‐Thiophenylacetic acid, 2,5‐dichlorophenyl ester (C_8_H_6_Cl_2_O_2_)	24.32	205.	3
17	6‐Nonyl‐5,6‐dihydro‐2H‐pyran‐2‐one (C_14_H_24_O_2_)	24.5	224.3	2.26
18	Hexadecanoic acid, ethyl ester (C_18_H_36_O_2_)	24.93	284.4	1.21
19	Hexasiloxane, tetradecamethyl‐(C_14_H_42_O_5_Si_6_)	24.98	458.9	3.23
20	2, 6, 10‐Trimethyltridecane (C_16_H_34_)	25.06	226.4	1.4
21	2‐(Pentadec‐14‐en‐1‐yl)furan (C_19_H_32_O)	25.21	276.5	0.57
22	13‐Tetradecen‐1‐ol acetate (C_16_H_30_O_2_)	26.66	254.4	2.16
23	11, 13‐Dimethyl‐12‐tetradecen‐1‐ol acetate (C_18_H_34_O_2_)	27.42	282.4	1.38
24	Heneicosane (C_21_H_44_)	28.75	296.6	1.8
25	D‐Homoandrostane, (5.alpha., 13.alpha.)‐ (C_20_H_34_)	29.14	274.5	0.43
26	5‐Isobutyl‐(13.alpha.H)‐isocopalane (C_24_H_44_)	31.43	332.6	0.67
27	L‐Histidine, N (1), N, N‐trimethyl‐, methyl ester (C_8_H_13_N_3_O_2_)	31.95	183.2	1.49
28	Pthalic acid, di (2‐propylpentyl) ester (C_26_H_26_O_4_)	34.17	402.5	13.69
29	Cyclopentanecarboxylic acid, hexadecyl ester (C_22_H_42_O_2_)	34.64	338.6	1.86
30	4H‐1,2,4‐triazole‐3,5‐diamine, N3‐(4‐fluorophenyl)‐N5‐methyl‐(C_9_H_10_FN_5_)	36.75	207.2	1.42
31	1,4‐Benzenedicarboxylic acid, bis (2‐ethylhexyl) ester (C_24_H_38_O_4_)	37.3	390.5	3.09
32	Arsenous acid, tris(trimethylsilyl) ester (C_9_H_27_AsO_3_Si_3_)	38.12	342.4	0.79
33	1,2‐Benzenedicarboxylic acid, dipropyl ester (C_14_H_18_O_4_)	38.42	250.2	2.01

**TABLE 5:** Phytochemicals detected in the extract of *Hordeum vulgare* by GCMS analysis.

Peak no.	Phytochemical	Retention time	Molecular weight	Area (%)
1	Benzothiazole (C_7_H_5_NS)	8.14	135	3.29
2	2‐Hexenoic acid, ethyl ester (C_8_H_14_O_2_)	14.25	147.9	2.28
3	2,4‐Di‐tert‐butylphenol (C_14_H_22_O)	14.48	286.2	6.71
4	1,2‐Butanediol, 1‐(2‐furyl) (C_8_H_12_O_3_)	18.78	123.1	5.64
5	3, 5‐di‐tert‐Butyl‐4‐hydroxybenzaldehyde (C_15_H_22_O_2_)	20.16	220.1	1.47
6	Hexadecanoic acid, methyl ester (C_17_H_34_O_2_)	23.6	74	4.01
7	Benzenepropanoic acid, 3,5‐bis(1,1‐dimethylethyl)‐4‐hydroxy‐, methyl ester (C_18_H_28_O_3_)	23.66	277.2	2.3
8	Oxalic acid, cyclohexylmethyl dodecyl ester (C_21_H_38_O_4_)	24.32	99.1	3.55
9	6‐Undecyl‐5,6‐dihydro‐2H‐pyran‐2‐one (C_22_H_40_O_2_)	24.49	99.1	3.18
10	Hexadecanoic acid, ethyl ester (C_18_H_36_O_2_)	24.92	88	3.63
11	9,12‐Octadecadienoic acid (Z,Z)‐, methyl ester (C_19_H_34_O_2_)	26.8	95	4.46
12	11‐Octadecenoic acid, methyl ester (C_19_H_36_O_2_)	26.93	95	3.27
13	Heptadecanoic acid, 16‐methyl‐, methyl ester (C_19_H_38_O_2_)	27.41	87	1.14
14	Linoleic acid ethyl ester (C_20_H_36_O_2_)	28.02	81.1	3.4
15	7‐Tetradecyne (C_14_H_28_)	28.13	95	2.48
16	trans‐beta. ‐Ionone (C_13_H_20_O)	28.67	177	2.38
17	Acetic acid, chloro‐, hexadecyl ester (C_18_H_35_ClO_2_)	28.88	97	1.95
18	Phthalic acid, di (2‐propylpentyl) ester (C_26_H_26_O_4_)	34.17	279	35.29
19	1,4‐Benzenedicarboxylic acid, bis (2‐ethylhexyl) ester (C_24_H_38_O_4_)	37.3	261.1	9.66

**TABLE 6:** Antimicrobial activity (zones of inhibition) (300 mL size of well) against *Pseudomonas aeruginosa*.

Names of the plants and drugs	Zones of inhibitions in “mm”
*Avena fatua* (Np3)	65
*Cirsium arvense* (Np4)	72
*Hordeum vulgare* (Np5)	60
*Chenopodium murale* (C.M)	52
*Convolvulus arvensis* (C.A)	61
**CIPRO1**	66
**CIPRO2**	62

**TABLE 7:** Values of MIC of the compounds having bacterial conc. 10^3^/mL.

Sr.	Compounds	MIC (in μg/mL)	MBC (in μg/mL)
1	*Avena fatua* (Np3)	1*	2*
2	*Cirsium arvense* (Np4)	2	4
3	*Hordeum vulgare* (Np5)	2	4
4	*Chenopodium murale* (C.M)	1*	2*
5	*Convolvulus arvensis* (C.A)	2	4

*Note:* The compounds marked with an asterisk showed the lowest minimum inhibitory concentration (MIC) and minimum bactericidal concentration (MBC) values, indicating a comparatively stronger antibacterial potential than the other tested compounds.

Ascorbic acid exhibited significantly higher DPPH free radical scavenging activity (% inhibition: 89.78%, 90.32%, 90.46%, and 90.73%) compared to the ethanolic extract of *C. arvensis*, which showed 71.66%, 80.92%, 85.01%, and 87.41% inhibition at corresponding concentrations of 5–20 mL, respectively. *C. murale* showed (78.33%, 63.48%, 54.09%, and 54.09%) inhibition at corresponding concentrations of 5–20 mL, respectively, *A. fatua* showed (27.24%, 5.18%, 5.05% and −9.81%) inhibition at corresponding concentrations of 5 mL, 10, 15 and 20 mL, respectively, *C. arvense* showed (78.88%, 81.61%, 83.24% and 84.33%), inhibition at corresponding concentrations of 5–20 mL, respectively, and *H. vulgare* showed (26.97, 2.45, 1.9%, and 1.63%). *Convolvulus arvensis* showed significantly higher inhibition of DPPH scavenging activity than the ethanolic extracts of *C. murale*, *A. fatua*, *Cirsium arvensis*, and *H. vulgare*.

FTIR analysis revealed the presence of functional groups, such as hydroxyl (—OH), carbonyl (C=O), ether (C–O), and sulfoxide (S=O), which are commonly associated with antioxidant mechanisms. These groups, particularly the O—H and C=O bonds, contribute to free‐radical scavenging by donating hydrogen or electrons. *Convolvulus arvensis*, which showed the highest antioxidant activity (87.41% at 20 μL), exhibited prominent peaks for these functional groups. This correlation supports the hypothesis that this observed DPPH activity is chemically driven by the bioactive moieties.

### 3.5 Sample Collection

Saliva samples were collected from Bakhtawar Amin Medical and Dental College, Multan.

### 3.6 Isolation of Bacterial Strain

**FIGURE 3:**
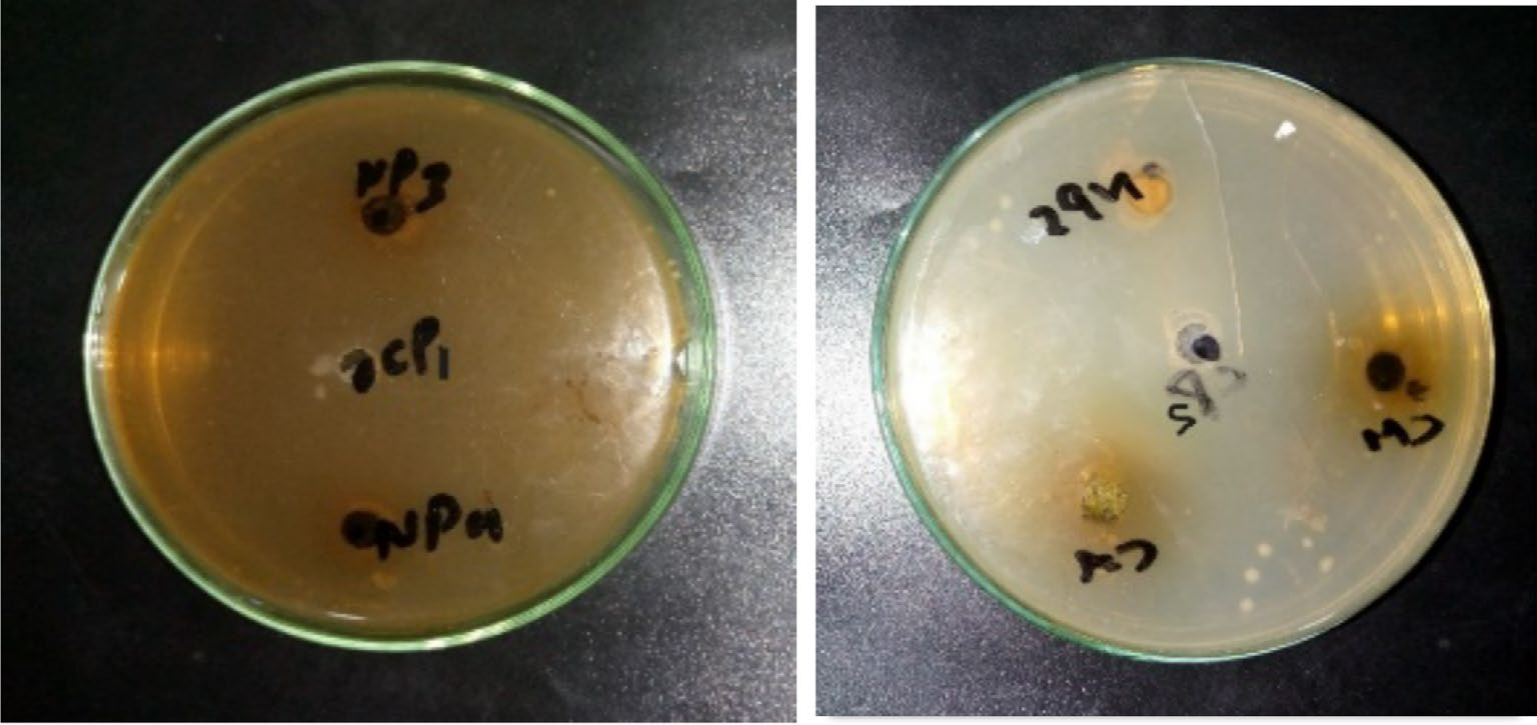
Zones around the extracts *Convolvulus arvensis*, *Chenopodium murale*, *Avena fatua*, *Cirsium arvense*, and *Hordeum vulgare* and CIPRO1, CIPRO2 drug against *Pseudomonas aeruginosa*.

Bacterial strains were isolated from saliva samples on a differential medium such as blood agar (Figure 3). The colonies were further purified on L‐agar.

### 3.7 Biochemical Characteristics of Bacterial Strain on Blood Agar

#### 3.7.1 Catalase Test

**FIGURE 4:**
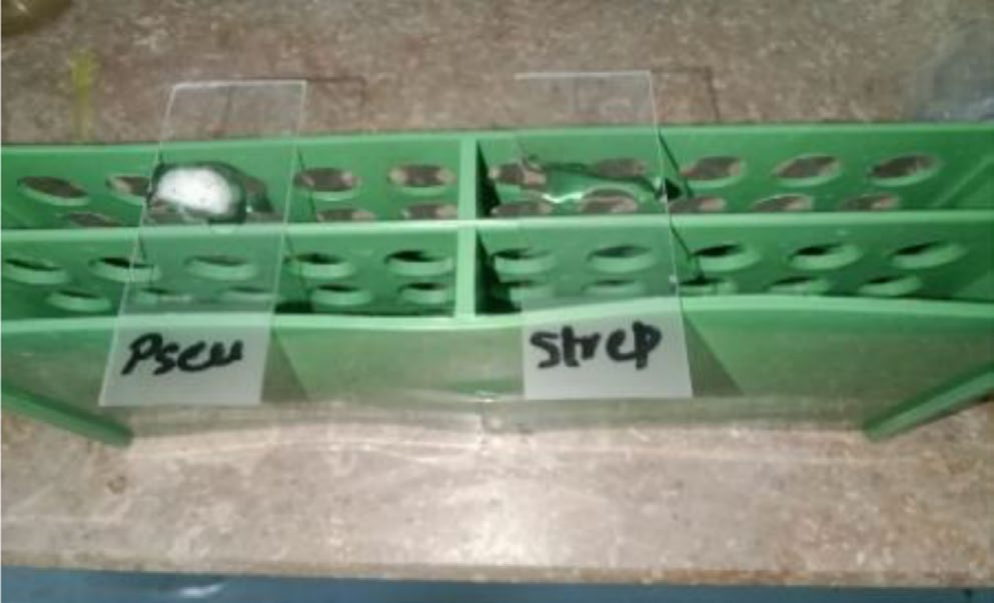
*Pseudomonas aeruginosa* gives catalase positive while *Strep. mutans* gives negative.

*Pseudomonas aeruginosa* gives a positive catalase test Figure 4 because it produces the catalase enzyme, which breaks down hydrogen peroxide into water and oxygen, resulting in bubble formation (Hyder et al. 2020; Paray et al. 2023).

#### 3.7.2 Gram Staining

**FIGURE 5:**
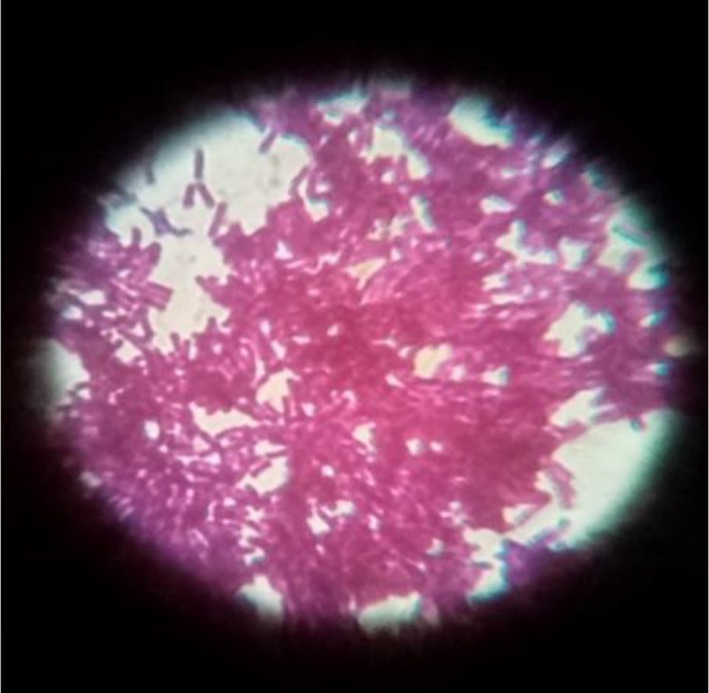
Gram‐negative rods of *Pseudomonas aeruginosa*.

As a gram‐negative bacterium, *P. aeruginosa* appears as pink or red rods under a microscope after Gram staining, as shown in Figure 5.

#### 3.7.3 Antibacterial Susceptibility Test

The inhibition zones of five plants extract *C. arvensis*, *C. murale*, *H. vulgare*, *C. arvense* and *A. fatua* against *P. aeruginosa* are 61, 52, 60, 72 and 65 mm, respectively. **CIPRO1** (ciprofloxacin 1) and **CIPRO2 (**ciprofloxacin 2), used as positive controls, exhibited zones of inhibition of 66 and 62 mm, respectively (Table 6). The *C. arvense* extract showed a higher zone of inhibition (72 mm) than the other extracts.

### 3.8 Minimum Inhibitory Concentration and Minimum Bactericidal Concentration

The MIC for *A. fatua*, *C. arvense*, *H. vulgare*, *C. murale*, and *C. arvensis* 1, 2, 2, 1, and 2 μg/mL, respectively, are presented in Table 7. Minimum bactericidal (MBC) concentrationsfor *A. fatua*, *C. arvense*, *H. vulgare*, *C. murale*, and *C. arvensis* 2, 4, 4, 2, and 4 μg/mL, respectively, are presented in Table 7.

*Avena fatua* and *C. murale* are effective at very low concentrations, indicating strong antimicrobial activity. They inhibit bacterial growth at 1 μg/mL and kill bacteria at 2 μg/mL.

**FIGURE 6:**
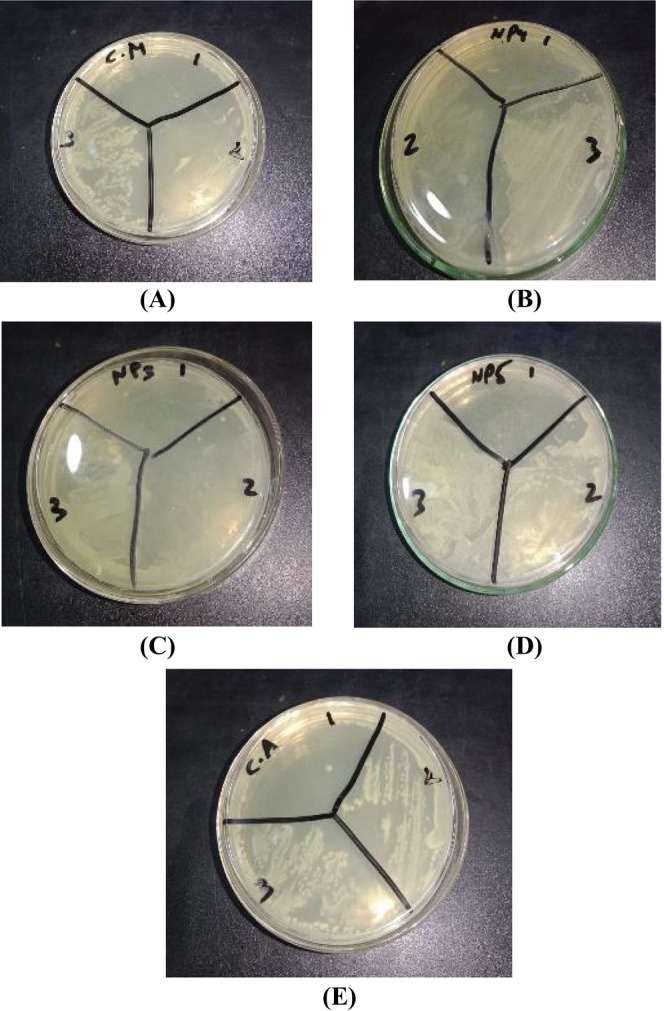
MIC value of plants against *Pseudomonas aeruginosa*: (A) *Chenopodium murale* (C.M), (B) *Cirsium arvense* (NP4), (C) *Avena fatua* (NP3), (D) *Hordeum vulgare* (NP5), and (E) *Convolvulus arvensis* (C.A).

*Convolvulus arvensis*, *H. vulgare*, and *C. arvense* require a slightly higher concentration to inhibit bacterial growth 2 and to kill bacteria at 4 μg/mL in Figure 6.

All antibacterial assays, including agar well diffusion and MIC and MBC evaluations, were conducted using identically prepared plant extracts and the same solvent throughout the study to ensure consistency. MIC and MBC were determined using serial dilutions of a standardized bacterial inoculum of approximately 10^3^ CFU/mL. MIC represents the lowest concentration of extract that prevents visible bacterial growth, whereas MBC is the concentration required to kill at least 99.9% of the bacterial population.

The values listed in Table 7 are visually supported in Figure 6. *Avena fatua* and *C. murale* demonstrated the strongest antibacterial activity, with MIC = 1 μg/mL and MBC = 2 μg/mL, respectively. *Cirsium arvense*, *H. vulgare*, and *C. arvensis* exhibited slightly weaker activities, requiring 2 μg/mL for MIC and 4 μg/mL for MBC. The figure legend has been updated accordingly to reflect the correlation between tabular and graphical data.

### 3.9 Biofilm Activity

**FIGURE 7:**
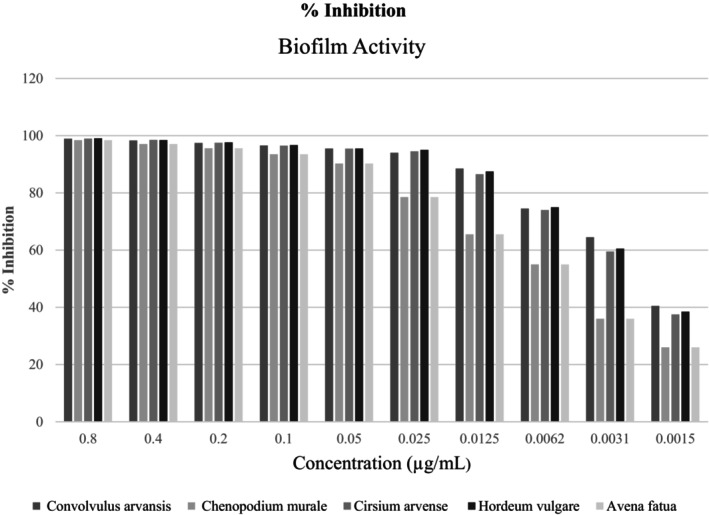
Biofilm activity of five plants.

In antimicrobial testing, inhibition percentages above 50% are considered significant, indicating substantial bacterial growth inhibition (Adeyemo et al. 2021). All studied plants were found to be effective in inhibiting biofilm formation by *P. aeruginosa*, but *H. vulgare* showed the highest percentage of inhibition at a concentration of 0.8 μg/mL. *Hordeum vulgare* appeared to be the most effective against *P. aeruginosa*, consistently showing high inhibition percentages across the tested concentrations, especially at 0.8 μg/mL. According to the classification criteria defined by Adeyemo et al. (2021), biofilm inhibition was categorized into three levels: strong (> 75%), moderate (50%–75%), and weak or no inhibition (< 50%). All five plant extracts exhibited strong biofilm inhibition at concentrations ranging from 0.8 to 0.05 mg/mL, with inhibition values exceeding 90%. However, noticeable differences were observed at concentrations below 0.05 mg/mL. *Hordeum vulgare* consistently maintained the highest inhibitory activity across all tested concentrations, whereas other plant extracts showed a sharper decline. This classification system, previously stated in the methodology, was applied here to improve the clarity and consistency between sections. *Convolvulus arvansis*, *C. murale*, *C. arvense*, and *A. fatua* also showed strong inhibition; however, *H. vulgare* showed slightly higher percentages at the highest concentration tested (Figure 7).

## 4 Discussion

This study focused on determining the antibacterial and antioxidant activities of five plant extracts (*C. arvensis*, *A. fatua*, *Cirsium arvensis*, *C. murale*, and *H. vulgare*) against *P. aeruginosa*. In contrast to our study, which used five plant extracts, different plant sections have been used in several studies. The bark of the Philodendron plant was used to prevent bacteria from growing in the mouth cavity. The bark of the Philodendron plant works well in mouthwashes and oral cleansers (Ai et al. 2025; Bencze et al. 2023). In one study, pomegranate and green tea extracts were synthesized using gamma radiation, while in our study, we used a shaker for three days after filtering. When used together, both plant extracts are effective against the oral bacterial inhibition zone (Ali et al. 2020).

The antioxidant qualities of ginger and its components have been studied. Ginger extract has been shown to have antioxidant properties in animals that have been tested (Ahmed et al. 2023). In our study, the antioxidant activities of the plant extracts were examined using DPPH scavenging activity. The percentage of inhibition of this extract varied. The zone of inhibition was less than that of the standard ascorbic acid. Among these five plants, *C. arvensis* showed a high zone of inhibition compared to the other four plants. These plant extracts can help stop dental cavities by being added to toothpaste, mouthwashes, and other medications. This study revealed that five plant extracts, including *C. arvensis*, exhibited significant inhibition zones against *P. aeruginosa. Cirsium arvense* extract showed the highest antimicrobial activity, with an inhibition zone of 72 mm. In the current study, different analyses of plant extracts, such as FTIR, which is crucial for identifying functional groups and bioactive compounds, were performed. Our plant extracts displayed various functional groups following FTIR analysis. Different functional groups, such as methylene, cyanide, and alcoholic groups, were also detected in another study using FTIR analysis of plant extracts of flax (*Linum usitatissimum* L.) (Saleem, Kamran, et al. 2020).

Phytochemical screening of the five medicinal plants revealed considerable diversity in the number and types of compounds present. Notably, 27 compounds were identified in *C. arvensis*, while *A. fatua*, *C. arvense*, *C. murale*, and *H. vulgare* contained 55, 24, 33, and 19 compounds, respectively. For example, identified key constituents identified include 1,2‐ethanediamine, N′‐ethyl‐N, N‐dimethyl‐ (36.01%), and 2,3‐butanediol, 1,4‐dimethoxy‐ in *C. arvensis*. Such chemical diversity suggests that these plants have significant potential as sources of bioactive compounds for therapeutic applications (Jalill et al. 2014; Luo et al. 2024).

In the current study, the antimicrobial activities of plants were examined with respect to antibiotics, using the agar well diffusion method. The inhibition zones of five plant extracts *C. arvensis*, *C. murale*, *H. vulgare*, *C. arvense*, and *A. fatua* against *P. aeruginosa* were 61, 52, 60, 72, and 65 mm, respectively. The extract of *C. arvense* extract showed a higher zone of inhibition (72 mm) than the other extracts. The potential of medicinal plant extracts to suppress or impede pathogenic growth in the oral cavity was the subject of numerous investigations, in contrast to ours. These studies used various portions of the extracts with anti‐biofilm and antibacterial properties against *S. mutans* and *C. albicans* (Long et al. 2025). When tested against *S. mutans*, the methanol extract showed strong antibacterial activity (> 80% growth suppression) (Duduk et al. 2018).

The MIC of *A. fatua*, *C. arvense*, *H. vulgare*, *C. murale*, and *C. arvensis* were 1, 2, 2, 1, and 2 μg/mL, respectively. *Avena fatua* and *C. murale* were effective at very low concentrations, indicating their strong antimicrobial activity. They inhibited bacterial growth at 1 μg/mL and killed bacteria at 2 μg/mL. *C. arvensis*, *H. vulgare*, and *C. arvense* required a slightly higher concentration to inhibit bacterial growth at 2 μg/mL and kill bacteria at 4 μg/mL. In a previous study, among the five isolates examined, cinnamon extract had an MIC value of 500 μg/mL against four isolates. The MIC values for various extracts against the isolates ranged from 500 to 2000 μg/mL. Although the MIC values appeared to be rather high, crude extracts may be effective. Contrary to our expectations, the limited potency of some extracts was also noted, even though a specific number of them showed good antibacterial potency. While a few extracts, such as cinnamon, neem, and mentha, have demonstrated strong in vitro efficacy against *P. aeruginosa* (Somsila et al. 2018).

Inhibition percentages above 50% were considered significant for antimicrobial testing, indicating substantial bacterial growth inhibition. In our study, all studied plants were found to be effective in inhibiting biofilm formation by *P. aeruginosa*, but *H. vulgare* showed the highest percentage of inhibition at a concentration of 0.8 μg/mL. *Hordeum vulgare* appeared to be the most effective against *P. aeruginosa*, consistently showing high inhibition percentages across the tested concentrations, especially at 0.8 μg/mL. *Convolvulus arvansis*, *C. murale*, *C. arvense*, and *A. fatua* also showed strong inhibition, but *H. vulgare* stood out with slightly higher percentages at the highest concentration tested (Oalđe Pavlović et al. 2024).

We agree that molecular confirmation would improve the accuracy of bacterial identification. In this study, classical morphological and biochemical criteria were thoroughly applied, including colony morphology, pigmentation (e.g., metallic green due to pyocyanin), beta‐hemolysis, distinct grape‐like odor, gram‐negative rod shape, and a positive catalase reaction. This approach aligns with the standard protocols for the preliminary identification of *P. aeruginosa*. However, we acknowledge that reliance on these methods alone, especially in clinical settings, may lead to misidentification even when selective media are used. Therefore, future studies should incorporate molecular diagnostic techniques, such as 16S rRNA sequencing or species‐specific PCR, to confirm bacterial species more reliably.

Although this preliminary study did not evaluate antibiotic resistance profiles or track clinical therapies and patient outcomes, the isolation of *P. aeruginosa*, a pathogen known for multidrug resistance, is of significant clinical interest. The strong antibacterial and anti‐biofilm activities exhibited by *A. fatua* and *C. murale* indicate their potential to combat resistant strains. In future investigations, we plan to incorporate antimicrobial susceptibility testing and patient‐level clinical data to better correlate the laboratory results with therapeutic outcomes.

A previous study demonstrated that cinnamon‐based water extracts limit oral bacterial production of exopolysaccharides and block the function of gtf enzymes. It also blocks acid release by bacterial biofilms, resulting in inhibition of caries development (Alshahrani and Gregory 2020).

## 5 Conclusion

This study revealed that plant extracts, including *C. arvensis*, exhibited significant inhibition zones against *P. aeruginosa. Cirsium arvense* extract showed the highest antimicrobial activity, with an inhibition zone of 72 mm. This study revealed that all studied plants were effective in inhibiting biofilm formation by *P. aeruginosa* but *H. vulgare* showed the highest percentage of inhibition at a concentration of 0.8 μg/mL. *Avena fatua* and *C. murale* were effective at very low concentrations, inhibiting bacterial growth at 1 and killing bacteria at 2 μg/mL. *C. arvensis*, *H. vulgare* and *C. arvense* require slightly higher concentrations to inhibit growth and kill the bacteria. Therefore, these plant extracts have proven to be beneficial in controlling oral diseases caused by *P. aeruginosa*.

## Author Contributions

**Nosheen Bibi:** conceptualization (equal), data curation (equal). **Shazia Perveen:** conceptualization (equal), data curation (equal). **Sumaira Kanwal:** conceptualization (equal), data curation (equal). **Fariha Latif:** methodology (equal). **Rehmana Rashid:** methodology (equal). **Sara Janiad:** formal analysis (equal). **Iram Qadeer:** formal analysis (equal). **Fatima Naseem:** writing – review and editing (equal). **Abdullah R. Alanzi:** writing – original draft (equal), writing – review and editing (equal). **Rashed N. Herqash:** funding acquisition (equal), resources (equal). **Imran Haider:** writing – review and editing; validation; funding acquisition. **Mehraj A. Abbasov:** resources (equal), software (equal). **Sadaf Kayani:** funding acquisition (equal). **Mohd Asif Shah:** Data curation, writing – review and editing.

## Ethics Statement

This study does not include human or animal subjects. This study was conducted in compliance with the ethical standards for human research. Prior to sample collection, all participants provided informed consent, and the research protocol was reviewed and approved by the Ethical Review Committee of The Women's University Multan. The study adhered to the Declaration of Helsinki and EU Directive 2001/20/EC guidelines for human experimentation. The official IRB approval number was WUM/REC/24‐20. Furthermore, this study did not involve any identifiable human data (such as images, names, or videos).

## Consent

The authors have nothing to report.

## Conflicts of Interest

The authors declare no conflicts of interest.

## Data Availability

All the data related to this work can be sourced from the corresponding authors.
